# MiR-125a-3p Regulates Glioma Apoptosis and Invasion by Regulating Nrg1

**DOI:** 10.1371/journal.pone.0116759

**Published:** 2015-01-05

**Authors:** Feng Yin, Jian Ning Zhang, Shu Wei Wang, Chun Hui Zhou, Ming Ming Zhao, Wen Hong Fan, Ming Fan, Shuang Liu

**Affiliations:** 1 Department of Neurosurgery, the Chinese PLA Navy General Hospital, Beijing, 100048, China; 2 Department of Brain Protection & Plasticity Research, Beijing Institute of Basic Medical Sciences, Beijing, 100850, China; Kyung Hee University, KOREA, REPUBLIC OF

## Abstract

The current study was designed to examine the functional role and mechanism of miR-125a-3p in glioma development. Quantitative RT-PCR was used to evaluate miR-125a-3p expression in 60 glioma cases of different malignant grades. Then, the clinic pathologic significance of miR-125a-3p expression was determined in combination with the prognosis of the patients. In addition, the effects and mechanisms of miR-125a-3p on the proliferation, apoptosis and invasion of glioma cells were further investigated. The results showed that the expression of miR-125a-3p was decreased significantly in most malignant glioma samples relative to normal brain tissues and glioma tissues of low-malignant degree. Further kaplan-meier survival analysis showed that the lower expression of miR-125a-3p was associated with a poor prognosis of GBM patients. Functional analysis showed that the reintroduction of miR-125a-3p into glioblastoma cell lines induces markedly the apoptosis and suppresses the proliferation and migration of glioblastoma cells in vitro and in vivo. Luciferase assay and Western blot analysis revealed that Nrg1 is a direct target of miR-125a-3p. Furthermore, an increased expression of Nrg1 could reverse the effects of overexpression of miR-125a-3p on the proliferation, apoptosis and migration of glioblastoma cells. These findings suggest that miR-125a-3p performed an important role in glioma development mediated by directly regulating the expression of Nrg1. This study also provides a potential target for diagnosis and treatment of malignant glioma.

## Introduction

Multiform glioblastoma and anaplastic astrocytomas are the most common malignant primary brain tumors in adults [1]. Although a great deal of effort has been devoted to finding a remedy during past decades, glioblastoma is still a deadly brain tumor with a median survival period of 14 months [2]. Malignant gliomas are aggressive because of their rapid proliferation and tendency to infiltrate brain tissue. Therefore, an investigation of the mechanism involved in the development and progression of glioblastoma is essential in order to find new therapeutic targets and an effective treatment strategy.

Alternations in miRNA expression have been implicated in the initiation, progression, and metastasis of a number of human cancers [3]. miRNAs consist of a class of small, non-coding RNAs (19–25 nucleotides) that function as post-transcriptional gene regulators [4–6]. They are primarily negative gene regulators of post-translational repression. Although the biological functions of most microRNAs have not been completely revealed, several studies have demonstrated that aberrant expression levels of microRNAs are involved in glioblastoma initiation and progression. It has been shown that microRNAs may function as oncogenes or tumor suppressor genes by regulating the expressions of the functional genes [7]. For example, miR-21 is highly overexpressed in glioblastoma and has important roles in cellular proliferation, invasion, and apoptosis by regulating the EGFR signaling pathway [8–10]. Important down regulated microRNAs have also been identified in glioblastoma, such as miR-124, miR-128 and miR-137[11–13]. In the current study, we focused on miR-125a-3p, which is generated as another strand of miR-125a-5p in the maturation process of precursor double strand RNA (pre-miRNA). Although many studies have shown that miR-125a-5p is an important tumor suppressor in many instances of tumors [14, 15], miR-125a-3p has been regarded as a passenger strand and has not been investigated extensively. However, recent reports indicate that miR125a-3p is also broadly conserved among vertebrates and may have cellular functions [16, 17]. However, there have no studies to date that demonstrate a contribution of miR-125a-3p to glioma progression. Further, how miR-125a-3p influences glioblastoma on a molecular basis is little understood.

According to the bioinformatics method, we have predicted that neuregulin 1(Nrg1) is a directed target of miR125a-3p. The influence of cytokines on tumor development has been studied for long time [18, 19]. Among cancer-related cytokines, Nrg1 is one of the most active members of the epidermal growth factor (EGF)-like family [20]. A lower level of Nrg1 expression is observed in more extensive brain areas of the adult brain, including the hypothalamus, hippocampus, basal ganglia, and brainstem [21, 22]. Nrg1 is the ligand of ErbB3 and ErbB4 [23]. Interaction by Nrg1 with the dimmers of its receptors leads to their activation. This further affects many biologic processes, including tumorigenesis of several cell types [24, 25]. Nrg1 has been shown to promote the invasive behavior of breast cancer cells and the motility and migration of human glioma cells [26–28].

In this study, we studied the expression and clinical significance of miR-125a-3p in the development of glioma for the first time. We also demonstrated that Nrg1, which has been implicated in transcriptional regulation, heterochromatin formation, genomic stability, cell-cycle progression and tumor progression, is a direct target of miR-125a-3p. We also provide evidence to show that miR-125a-3p inhibits glioma cell proliferation and invasion both in vitro and in vivo by regulating Nrg1. This study suggests that miR-125a-3p may be a critical therapeutic target for glioma intervention.

## Materials and Methods

### Tissue specimens and patient information

The tumor samples were obtained from patients who were undergoing surgical treatment at the Navy General Hospital during the period of 2002 to 2006. These tumors were classified as different glioma subtypes, according to World Health Organization (WHO) criteria. Twenty of these gliomas were glioblastoma (GBM, WHO grade IV), 20 were anaplastic astrocytoma (AAST, WHO grade III), and 20 were astrocytoma (AST, WHO grade II). Normal human brain tissues that were obtained from five patients who suffer from epilepsy were used as controls. This study was approved by the institutional review boards of the Navy General Hospital of PLA. All specimens were flash-frozen in liquid nitrogen after resection. Written informed consent to use the sample in future research was obtained from each patient. The purity of each tumor sample was estimated by hematoxyalin and eosin (HE) staining to ensure that it contained more than 70% neoplastic cells.

### Animals

The Animal Center of Chinese Academy of Medical Science supplied 30 female athymic BALB/c nude mice (female), which weighed from 25g to 28g. The mice were bred in laminar flow cabinets under specific pathogen-free conditions and handled according to the license of laboratory animal environment and facilities issued by China (GB1425-1010).

### Statement of ethics

We obtained written informed consent from all participating patients who were informed of, and understood, the purpose and risk in providing specimens. This study was approved by the Medical Ethics Committee of Navy General Hospital, PLA, Beijing, China. The animal study was performed in strict accordance with the recommendations in the Guide for the Care and Use of Laboratory Animals of the Chinese Institute of Health. The protocol was approved by the Committee on the Ethics of Animal Experiments of Navy General Hospital (Permit Number: 0308-2013). All surgery was performed under sodium pentobarbital anesthesia, and every effort was made to minimize suffering.

### The expression of miR-125a-3p in clinical glioma samples

Total RNA was isolated from tissues and cell lines using Trizol reagent (Invitrogen) for miR-125a-3p and Nrg1 analyses according to the manufacturer’s protocol. For miR-125a-3p and Nrg1 expression analysis, total RNA was retro transcribed with microRNA-specific primers (3’-GATGCTCTACAGGTGAGGTTCTT-5’) and Nrg1 primers (FP: 5′-CGTGGAATCAAACGAGATCATCA-3′ and RP: 5′-GCTTGTCCCAGTGGTGGATGT-3′) using TaqMan microRNA and an mRNA reverse transcription kit (Applied biosystems,Foster City, CA), Then, quantitative RT-PCR (qRT-PCR) was performed using Tagman microRNA and mRNA assays according to the manufacturer’s protocol. The comparative cycle time (Ct) method was used to calculate the relative abundance of miR-125a-3p and Nrg1 in comparison to the expressions of RNAU6B small nuclear RNA and β-actin [29].

### Cell lines and primary cell culture

Established glioblastom cell lines U251and U87-MG were acquired from the American Type Culture Collection. Cells were cultured in DMEM/F12 supplemented with 10% fetal bovine serum at 37°C in a humidified 5% CO_2_ incubator. The cell count and viability were determined by Trypan Blue staining.

### Oligonucleotide, Nrg1-AAV2 expression vector synthesis, and transfection

MiR-125a-3p mimics (sence: ACAGGUGAGGUUCUUGGGAGCC and antisense: CUCCCAAGAACCUCACCUGUUU) and negative control (scrambled oligos) (GenePharma, Shuanghai, China) were transfected into glioblastoma cell lines using Lipofectamine 2000 (Invitrogen) at a final concentration of 100nM. Then, stable clones were generated by G418 selection. To generate a recombinant AAV serotype 2-Nrg1(rAAV2-Nrg1) viral vector, full-length cDNA for human Nrg1 was obtained by EcoRI and BamH1 digestion and sub cloned into the pSNAV plasmid (Invitrogen). It was then recombined into rAAV2 (Vector gene technology of Beijing, China). U251 and U87 MG cells were infected with rAAV-Nrg1 or control virus to generate Nrg1-overexpressing glioblastoma cells. The efficiency of infection was 90% (moi = 500).

### Proliferation assay

2×10^3^ glioblastoma cells per well were placed onto a 96-well plate at 2000 cells/well. After the cells were transfected with miR-125a-3p mimics and negative controls for 24, 48 and 72 hours, CCK-8 reagent was added to the cells. The cells then were further cultured in the chamber for two hours. Then, the optical density (OD) of 450 nm was measured by a microplate reader according to the manufacturer’s instructions.

### Apoptosis assay

Apoptosis was assessed by measuring the membrane redistribution of phosphatidylserine with fluorescent annexin V. The cells were collected, and washed twice with PBS and then re-suspended in 500μl of a staining solution that contained 5μl of FITC-conjugated annexin V antibody (BD Pharmingen) and 10μl of propidium iodide (BD PharMingen). Immediately following incubation for 15 min at room temperature in the dark, the cells were analyzed on a flow cytometer. A data acquisition analysis was conducted in an FACS caliber flow cytometer (Becton Dickinson) using CellQuest software. Apoptotic cells were positive staining of annexin V and negative staining of propidium. The percentage of cells undergoing apoptosis was determined in three independent experiments. The apoptotic cell death in tumor specimens of nude glioblastoma model was examined by the TUNEL method using an in situ cell death kit (Roche, Indianapolis, IN, USA). We conducted the experiment according to the manufacturer’s instructions. The reaction mixture was incubated without enzyme in a control coverslip to detect nonspecific staining. DAB substrate was used to convert the fluorescence signal. Then, the positive cells were visualized under a light microscope.

### Monolayer wound scraping assay

Migration ability was determined by a wound scraping assay. Glioblastom cells overexpressed with indicated vectors which were indicated previously were grown in 6-well culture plates containing DMEM with 10% FBS. After the cells reached a 90% confluence, the medium was replaced by FBS-free medium for 24h. A sterile 200-μl pipette tip was used to create a wound in the monolayer by scraping. The cells were washed with PBS and grown in an FBS-free medium for 24h. The wounds were observed under a phase contrast microscope (model BX2; Olympus). The width of the scratch was measured at 0 and 24 h after treatment. The migration rates of the glioblastoma cells overexpressed of indicated vectors were calculated according to the following formula: (cell-free area at 0 h – cell-free area at 24 h)/ cell-free area at 0 h. Experiments were performed three times in duplicate with comparable results. The value are shown as mean ± SD.

### Transwell invasion assay

Glioblastoma cells were introduced by indicated vectors as described previously and cultured for 48 hr. Then they were transferred to the top of Matrigel-coated invasion chambers (24-wells insert, 8μm pore size; BD-Becton, Dickinson) according to the manufacturer’s protocol. After incubating at 37°C for 30min, the invasive cells that attached to the lower surface of the membrane insert were fixed in 100% methanol at room temperature for two min and stained with crystal violet 0.1% (Amreco, USA) before being counted under an inverted microscope. The experiments were performed in triplicate in three independent sets. The values are shown as mean ± SD.

### Nude mouse tumor xenograft model

30 nude mice were randomly assigned to three groups with 10 mice to each group. The U87 glioblastoma cell line was transfected with scramble oligo, miR-125a-3p mimics and infected with AAV2-Nrg1 separately. Then, the tumor cells were labeled as U87-scramble, U87-miR-125a-3p and U87-miR-125a-3p+Nrg1. Then, they were suspended in 4μl of normal saline. The solution was injected into the right caudate nucleus of the nude mice in three groups separately through a glass electrode connected to a Hamilton syringe under the guidance of a stereotactic system. The injection site was 2 mm lateral and 1 mm anterior to the bregma; a depth of 2.5 mm from the dura. The injection speed was 0.2μl/min. To study the kinetics of glioma cell growth in vivo, U87 cells (3×10^6^ cells in 50μl of PBS) with the different labels described above were injected subcutaneously into the right armpit of these nude mice concurrently. The nude mice were sacrificed after one month of intracranial transplantation or when they became moribund or showed signs of obvious neurological deficit. The paraffin sections (4μm) of the xenografted tumors were analyzed by H&E staining and immunohistochemical staining.

### Immunohistochemistry experiment

Endogenous peroxidase was neutralized with 3% H2O2 in methanol (10min) after antigen retrieval in a 0.1M critrate buffer (PH5.8) at 95°C for 5min and cooled at 25°C for 1h. Sections were blocked with normal human serum albumin (10min), then treated with the following primary antibodies overnight at 4°C: Rabbit anti-human Nrg1 polyclonal antibody(1:1000, Santa cruz, Texas); Goat anti human Ki67 monoclonal antibody ((1:1000, Santa cruz, Texas). After treatment with biotinylated secondary antibody, color reactions were performed with diaminobenzidine (DAB) (Sigma) and counterstained with Mayer’s hematoxylin.

### Protein extraction and western blot analysis

Total protein was extracted by using NP40 lysis buffer (0.5% NP40, 250-mM NaCl, 50mM HEPES, 5mM ethylenediaminetetraacetic acid, 0.5mM egtazic acid) supplemented with protease inhibitor cocktails (Sigma-Aldrich, St. Louis, Missouri). Lysates were subjected to centrifugation at 12,000 rpm for 10min, and the supernatant was collected for use in experiments. Protein lysates (40 μg) were resolved on denaturing sodium dodecyl sulfate-polyacrylamide gels ranging from 4% to 20% and transferred to nitrocellulose membranes (Bio-Rad Laboratories, Hercules, CA). The membranes were probed with the following antibodies: Rabbit anti-human Nrg1 polyclonal antibody (Abcam, UK) and then secondary antibody labeled by horseradish peroxidase (Amersham GE Healthcare, Chalfont St Giles, United Kingdom). The secondary antibody was visualized by using the ECL chemiluminescent reagent kit (Amersham GE Healthcare).

### Luciferase assay and vector construction

For the luciferase assay, the region that was predicted to harbor interacted sites 7-29bp of Nrg1 3’UTR (NM_13964) was amplified by PCR from human genomic DNA and inserted into the psiCHECK-2 vector (wild-type) (Promega, Madison, Wisconsin). The mutant type was generated with deletions by replacing the binding site of miR-125a-3p with the restriction enzyme cutting site AACGTGA. U87 was plated in 96-well dishes at 10^4^ cells per well. The U87 cells were co-transfected with miR-125a-3p and psiCHECK-2 Nrg1 3’UTR constructs (wild-type) (100μM) or scrambled with psiCHECK-2 Nrg1 3’UTR constructs (mutant) with the use of Lipofectamine 2000. Twenty-four hours after transfection, the cells were incubated for 10 min with 20μl/well 1×Passive Lysis Buffer (Promega). Firefly and renilla luciferase activities were measured sequentially using dual-luciferase assays (Promega) by a Veritas microplate luminometer (Turner BioSystems, Sunnyvalc, CA). The experiments were performed in quadruplicate in three independent sets. The values are shown as mean ± SD.

### Statistical analysis

The results were analyzed by Student’s two-tailed/test (P<0.05 was considered to be significant). All statistical analyses and graphing were conducted by the SPSS 12.0 Windows version of software (SPSS).

## Results

### Decreased expression of miR-125a-3p and increased expression of Nrg1 in gliomas were associated with a poor prognosis of glioma patients

**Figure 1:**
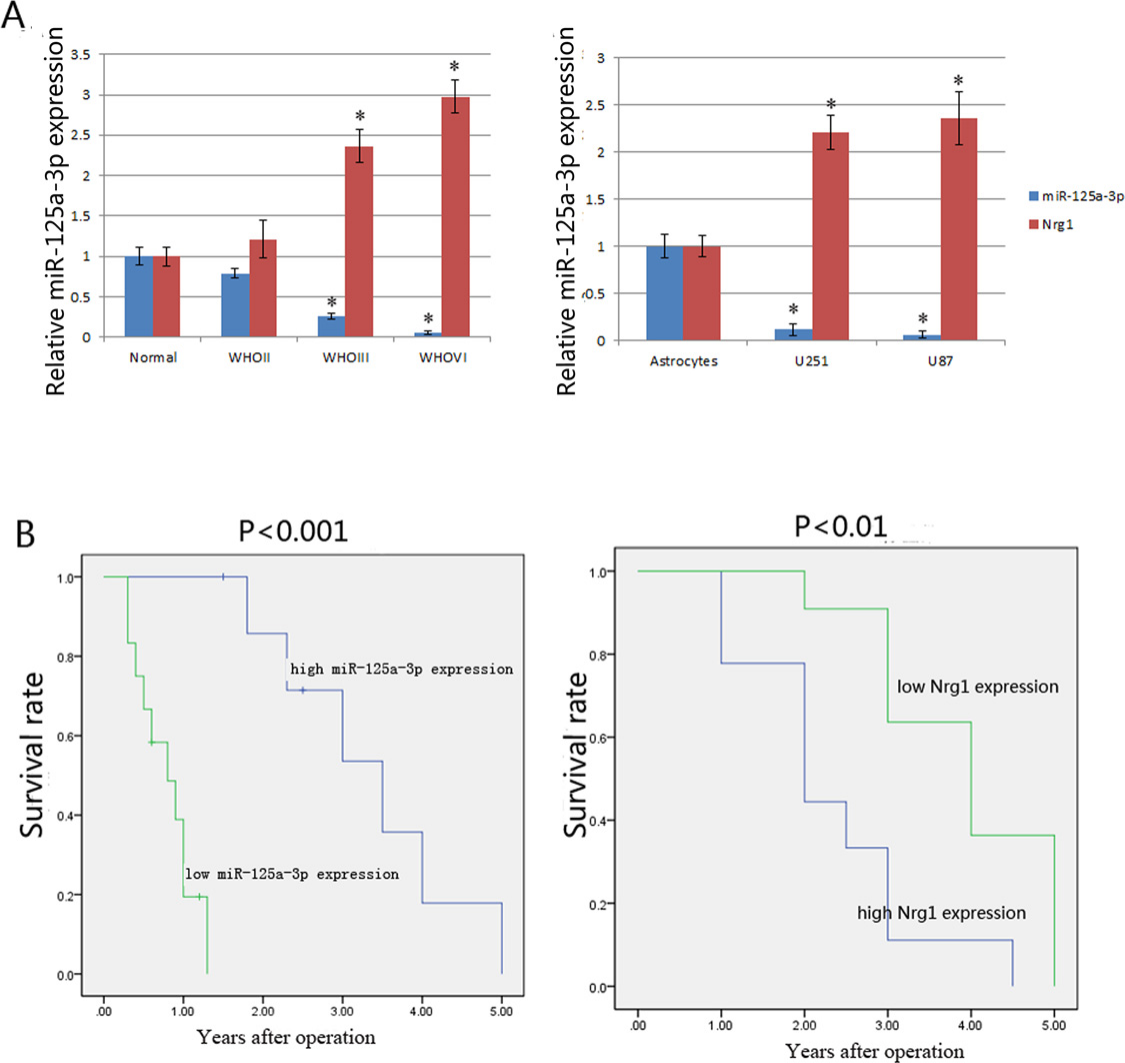
The expressions of miR-125a-3p and Nrg1 in glioma development and the relationship between their expressions and the survival time of glioma patients. A) Using qRT-PCR method, the expressions of miR125a-3p and Nrg1 in glioma tissues of different grades and normal brain tissues derived from epilepsy patients (left), as well as their expressions in normal astrocytes and glioblastoma cell lines (right), were analyzed. B) Kaplan-Meier overall survival curves of glioma patients according to the levels of miR125a-3p expression (left) and Nrg1 expression (right). The high miR-125a-3p expression group (n = 8) and the low miR-125a-3p expression group (n = 12); the high Nrg1 expression group (n = 10) and the low Nrg1 expression group (n = 10).

The expression level of miR-125a-3p was significantly higher in normal brain tissues than in glioma tissues, and decreased with an increase in the malignant grades of glioma. (Fig. 1A). The 20 glioblastoma cases were classified into two groups according to the median ration of miR-125a-3p expression level (6.538, normalized to U6) that was determined by qRT-PCR. Eight cases were assigned to the high expression group and 12 to the low expression group. An analysis of the 5-year overall survival curves showed that the patients in the low miR-125a-3p expression group had a significantly poorer prognosis than those in the high expression group (p<0.05) (Fig. 1B, left). Then, we detected the expressions of miR-125a-3p in normal astrocytes and glioblastoma cell lines. The results showed that the expression of miR-125a-3p was significantly lower in glioblastoma cell lines (U251 and U87) than in normal astrocytes (Fig. 1A). Furthermore, the study also showed that the expression of Nrg1 in glioma specimens was negatively correlated with the expression of miR-125a-3p (Fig. 1A), and that there was a positive correlation between the expression of Nrg1 and the survival time of the same cohorts of patients (p<0.01) (Fig. 1B, right).

### Restoration of miR-125a-3p in glioblastoma cells inhibited cellular migration and invasion

**Figure 2:**
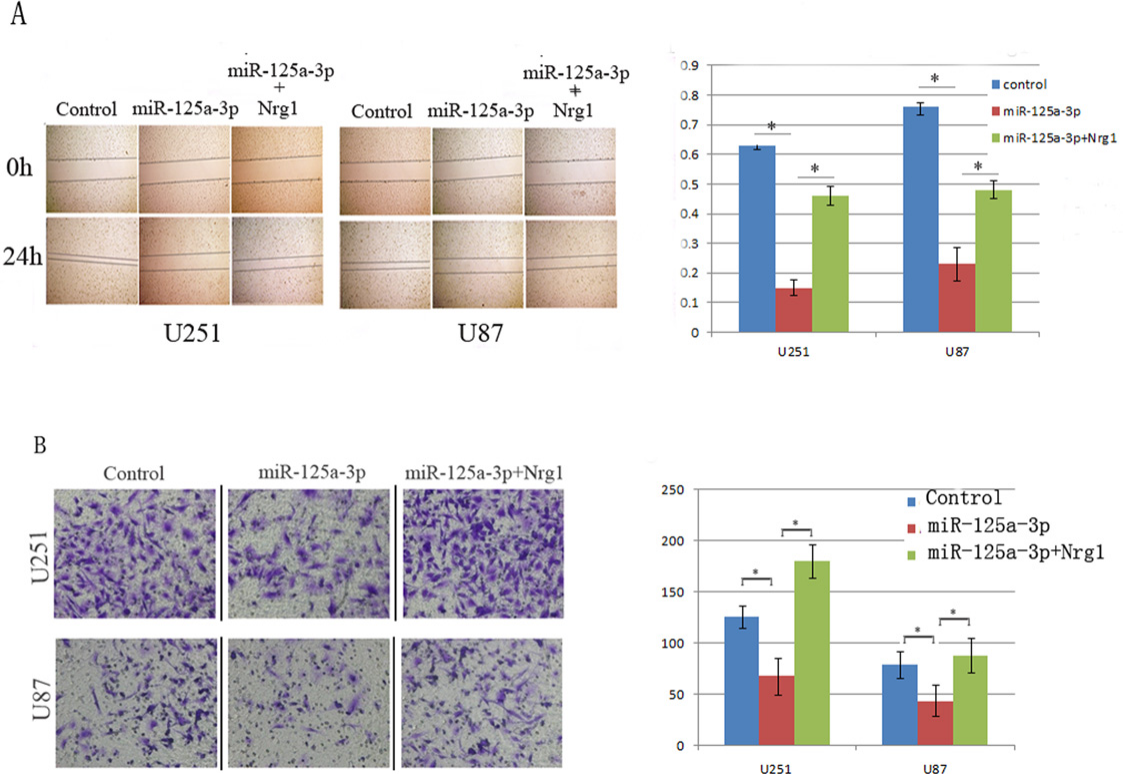
Effects of miR125a-3p on the migratory ability of glioblastoma cell lines. A) Wound scraping assay results showed that the distance of migration in U251 and U87 cells were significantly decreased after transfection of miR-125a-3p mimics, and that Nrg1 overexpression could increase the migration distances of glioblastoma cells transfected with miR-125a-3p. B) A Transwell assay showed that the numbers of U251 and U87 cells that migrated through a microporos membrane were significantly decreased after transfection of miR-125a-3p mimics, and that Nrg1 overexpression could eradicate the effect of miR-125a-3p in inhibiting invasion. All experiments were performed in triplicate in three independent sets. The values are shown as mean ± SD.

To determine if miR-125a-3p could modulate the migration and invasiveness of glioma cells, the U251 and U87 glioblastoma cell lines that expressed lower levels of miR-125a-3p (Fig. 1A, right) were selected for further study. We first examined the effect of miR-125a-3p on cell migration using the wound-scraping assay. As shown in Fig. 2A, compared to the control-vector transfected cells, which almost spread to the center line within 24 h, miR-125a-3p mimics-transfected cells exhibited considerably slower migration and decreased cell spreading. To further determine the effect of miR-125a-3p expression on the motility of glioma cells, a Transwell penetration assay was conducted. As shown in Fig. 2B, fewer miR-125a-3p mimics transfected cells invaded across the membrane pre-coated with Matrigels than the control cells. Taken together, these observations suggested that miR-125a-3p strongly inhibits the migration and invasion of glioma cells.

### miR-125a-3p induces apoptosis and inhibits proliferation in glioblastoma cells

**Figure 3:**
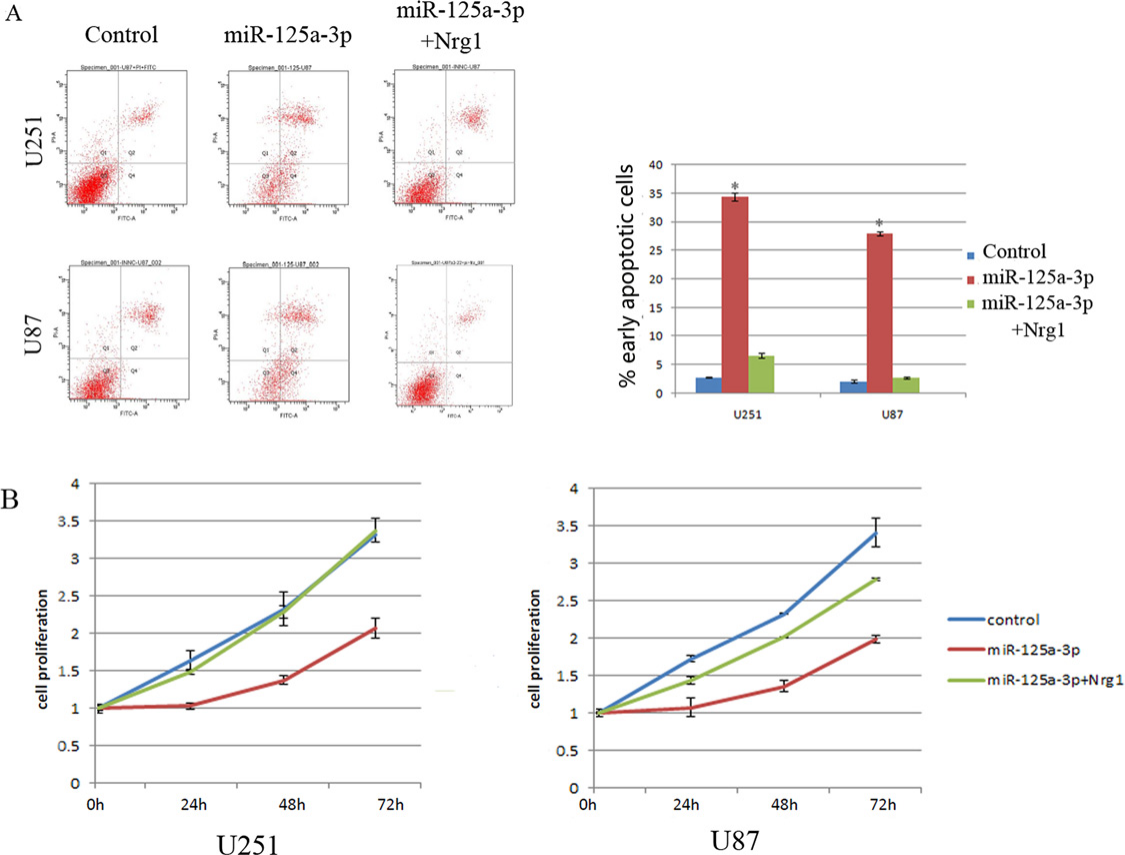
miR125a-3p induces apoptosis and inhibits proliferation of glioblastom cells. A) An Annexin V assay was performed 48 hrs. after transfection of U251 and U87 cells with miR125a-3p mimics, scrambled and further infected with Nrg1 expression plasmid (left). A statistical analysis of the numbers of apoptosis cells of above groups (right), B) The ability of glioblastoma cells to proliferate was detected by CCK8 assay. All experiments were performed in triplicate in three independent sets. The values are shown as mean ± SD. * represents a significant difference (p<0.05) by student’s t test.

Annexin V assay showed that miR-125a-3p mimics could significantly increase the numbers of early apoptosis in U251 and U87 cells lines compared to negative controls (Fig. 3A). CCK-8 assay showed that miR-125a-3p was able to reduce the proliferation of glioblastoma cells in a time-dependent manner (Fig. 3B).

### Reintroduction of miR-125a-3p inhibited tumorigenecity and invasion of glioblastoma cells in vivo

**Figure 4:**
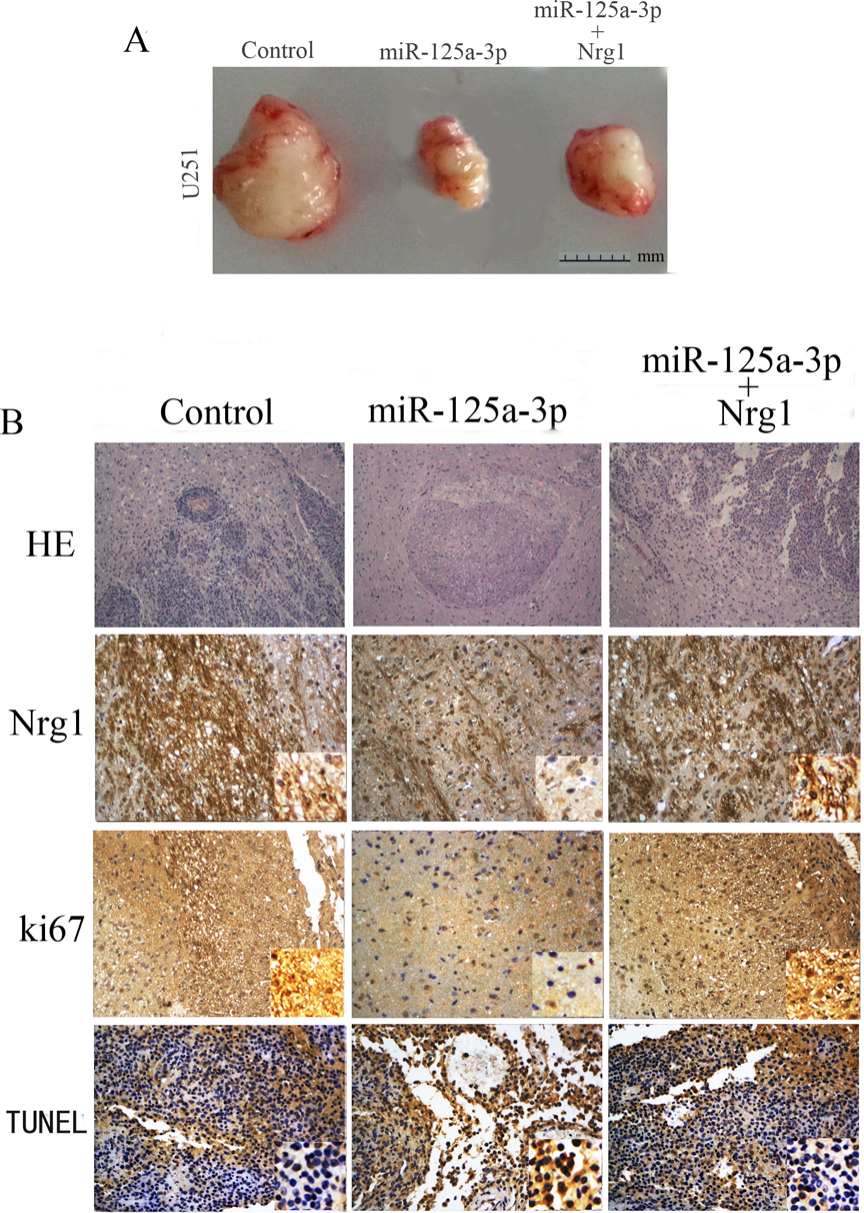
Reintroduction of miR125a-3p suppresses glioma tumorigenesis and invasiveness *in vivo*. A) The tumor mass raised from glioblastoma cells that were transfected with miR-125a-3p mimics was significant smaller than those transfected with control vectors. B) H&E staining analysis showed that transfection of miR-125a-3p mimics could significantly inhibit the invasion of intracranial glioblastoma, and that this effect of inhibition on invasion could be blocked by Nrg1 overexpression. The immunohischemical (IHC) analysis showed that miR-125a-3p mimics effectively inhibited the expression of Nrg1, and down-regulated the expression of Ki67. A TUNEL assay in xenograft tumor sections revealed that miR-125a-3p induced apoptosis of intracranial glioblastomas. Furthermore, overexpression of Nrg1 could block above expressional changes in intracranial glioblastomas.

In order to determine whether miR-125a-3p is involved in the tumorigenesis and invasiveness of glioma cells in vivo, we employed a subcutaneous xenograft and an intracranial xenograft in the nude mouse model system. As shown in Fig. 4A, the tumor mass raised from U87 cells that were transfected with miR-125a-3p was significantly smaller than that in glioma cells that were transfected with control vectors. Furthermore, an H&E staining assay showed that intracranial tumors formed by U87 cells that were transfected with control vectors exhibited an extensive branch-like growing pattern that spread into the surrounding tissue. In contrast, miR-125a-3p mimics–overexpressing U87 cells did not result in the formation of invasive tumors, instead of delimited lesions (Fig. 4B). The further immunohistochemistry experiments showed that miR-125a-3p overexpression could down-regulate the expression of Nrg1 (Fig. 4B). In addition, Ki67 staining showed that the U87-miR-125a-3p tumors had a lower proliferation index than the U87-miR-125a-3p scramble group. TUNEL assay analysis of xenograft tumor revealed more apoptosis in the U87-miR-125a-3p group than the tumors from the U87-miR-125a-3p scramble group (Fig. 4B).

### Nrg1 is a direct target of miR-125a-3p

**Figure 5:**
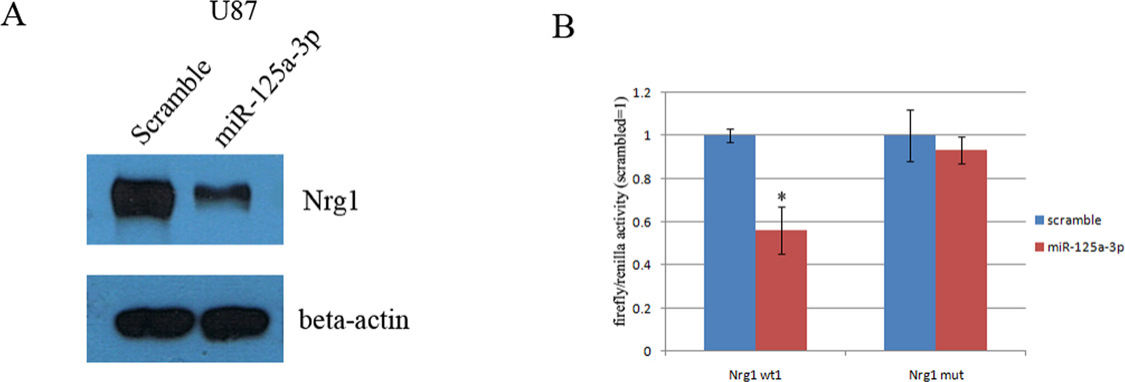
MiR125a-3p directly targets the Nrg1 3’UTR in U87 glioblastoma cell line. A) Western blotting for Nrg1 at 48 hrs. after transfection of miR125a-3p mimics in U87 cells. B) Double luciferase reporter assays confirmed that Nrg1 is the direct target of miR125a-3p in U87 cells. *P<0.05.

We predicted target genes of miR-125a-3p using multiple bioinformatics software and found that the 3’UTR of Nrg1 gene contains highly conserved regions that may serve as binding sites for miR-125a-3p. Then, Western blot analysis showed that miR125a-3p overexpression could decrease the expression of Nrg1 in the U87 glioblastoma cell line (Fig. 5A). To further demonstrate that Nrg1 is a potential target of miR-125a-3p, we generated luciferase reporters that contained the 3’UTR of the Nrg1 gene. The results of three independent experiments showed that reporter activity was reduced by the ectopic expression of miR-125a-3p (p<0.001, Fig. 5B). We also generated luciferase reporters that contained a mutated sequence within the predicted target sites of the 3’UTR of the Nrg1 gene (Fig. 5B) to further demonstrate the interaction between miR-125a-3p and the 3’UTR of Nrg1. The data showed that the reporter activity was not reduced by the ectopic expression of miR-125a-3p (Fig. 5B).

To confirm that the phenotypes induced by miR-125a-3p were mediated by Nrg1, we further over-expressed Nrg1 that did not contain the 3’UTR in miR-125a-3p transfected U87 cells. As shown in Figs. 2, 3 and 4, restored Nrg1 expression could significantly abrogate the effects of miR-125a-3p on invasiveness, apoptosis and the promotion of glioblastoma cells.

## Discussion

In this study, we provided the first evidence that the expression of miR-125a-3p was decreased significantly in malignant glioma tissues and glioblastoma cell lines, and reported that the lower expression of miR-125a-3p in glioma was associated with poor clinical prognosis (Fig. 1). The extremely poor prognosis of patients with gliomas is largely due to the high tendency of tumor invasiveness, unlimited proliferation, and abnormal regulation of cell apoptosis that lead to severe structural and functional damage to the surrounding brain tissue and occurrence and progression of tumor [30]. As a result of our present study, we have made clear that miR-125a-3p down-regulation in malignant glioma contributes to these tumor-specific behaviors in glioma cells. It is interesting that, the proliferation and invasion of glioblastoma cells were found to be inhibited, and that the apoptosis was found to be induced by restoration of miR-125a-3p in vitro and in vivo (Figs. 2, 3, 4). This led us to further study the mechanisms of miR-125a-3p that are acting on glioblastoma cells.

MiRNAs may negatively control downstream targeted genes to interfere with cell activities. Therefore, the expressions and functional roles of miRNAs are very important in tumor development. MiRNAs have been shown to negatively regulate the post-transcriptional expression of their target genes, which include tumor suppressors and oncogenes [7]. For example, miR-21 was found to be highly expressed in colorectal cancers, and induced metastasis and invasion by inhibiting the tumor suppressing gene PDCD4 in colorectal cancer cells [31]. Thus, as an oncogene, miR-21 exhibited regulatory effects in many instances of cancer development. However, when expressed at lower levels in tumor, miRNAs also demonstrated tumor suppressing functions. For example, the action of let-7 miRNA as an antioncogene was found to be absent in lung cancers and to promote cancer progression by inhibiting downstream translation of MYC [32]. MiR-125a-3p, a member of the miR-125a family, is derived from the 3’-end of pre-miR-125a. It has been reported that MiR-125a controls the differentiation of the embryonic cancer cell line P19 by negatively regulating the 3’UTR of lin-28 mRNA [33]. It was also found to inhibit translation of the target gene t-trkC, thereby modulating proliferation of neuroblastomas [34]. MiR-125a-5p, the partner of miR-125a-3p, was found to be down-regulated in breast cancer [35–37], ovarian cancer [38], lung cancer [39], and medulloblastoma [40], thus indicating its tumor suppression effect in human cancers. However, the reports of the new member of miR-125a-3p are still limited and vague. The expression of miR-125a-3p has been shown to be up-regulated in synovial sarcoma [41], and overexpressed under hypoxic conditions in retinoblastoma [42]. In contrast, it is reportedly down-regulated in non-small cell lung cancer [43]. Although, Lihi et al. reported that miR-125a-3p reduces proliferation and migration of HEK293T cells by targeting Fyn, they did not study the expression and functional roles of miR-125a-3p in glioma tissues and glioblastoma cell lines. So, there are still no reports of miR-125a-3p in the development of glioma.

It has been shown that cytokines affect tumor cell behavior for a long time [44, 45]. Nrg1 is one of the most active members of the epidermal growth factor (EGF)-like family [46], and also is considered to be a cancer related cytokine. The study by Sheng et al. showed that the ErbB3/Nrg1 autocrine loop supports the proliferation of ovarian cancer cells [47]. As in as our study (Fig. 1), Zhao et al. found that the expression of Nrg1 tended to increase with increasing WHO glioma grades, and contributed to malignancy by enhancing their migration [48]. Liu et al. reported that activation of ErbB receptors could promote human glioma cell motility and migration [49].

In the DIANA microT-CDS database, miR-125a-3p is shown to have multiple predicted targets among which there are many tumor suppressors. It has been reported that among these tumor suppressors, Nrg1 is dysregulated in glioma and could promote human glioma cell motility and migration [49]. Furthermore, we confirmed that the expression of Nrg1 could be down regulated by the overexpression of miR125a-3p in glioblastoma cell lines (Fig. 5A). Therefore, we concluded that Nrg1 may play an important role in mediating miR-125a-3p functions in glioma development. In addition, as a result of a double luciferase assay, we found that miR-125a-3p inhibits Nrg1 expression in glioblastoma cells by binding to the 3’UTR of Nrg1. In miR-125a-3p transduced cells, the ectopic expression of Nrg1 is able to abrogate most effects of migration inhibition and apoptosis promotion in glioblastoma cells (Figs. 2, 3, 4). This confirmed that miR-125a-3p may regulate the development of glioblastoma in an Nrg1-dependant manner. It also confirmed that Nrg1 is a major target of miR-125a-3p and is responsible for regulating invasion and apoptosis in human glioma.

Together, this study provides new insights into the role of miR-125a-3p in human gliomas. It has shown that miR-125a-3p is down-regulated in malignant gliomas and that miR-125a-3p overexpression potently inhibits glioma growth and invasion by targeting a tumor suppressor-Nrg1. The study also suggests that miR-125a-3p might serve as a target for glioma therapy.
